# Resting-state EEG reveals slowing and altered functional connectivity in children and young adults with severe chronic kidney disease

**DOI:** 10.1016/j.cnp.2026.06.002

**Published:** 2026-06-12

**Authors:** S. Lijdsman, M. Königs, M.S. van Sandwijk, A.H. Bouts, K. van Hoeck, H. de Jong, H. Bruining, F.J. Bemelman, A.F. van Rootselaar, K.J. Oostrom, J.W. Groothoff, C.A. Bosman

**Affiliations:** a Emma Children's Hospital, Amsterdam University Medical Centers (Amsterdam UMC), University of Amsterdam, Psychosocial Department, Amsterdam, the Netherlands, G8-136, PO Box 22660, 1100, DD, Amsterdam, the Netherlands; b Amsterdam Reproduction and Development research institute, Amsterdam, the Netherlands; c Emma Children's Hospital, Amsterdam UMC, University of Amsterdam, Emma Neuroscience Group, Department of Pediatrics, Amsterdam, the Netherlands; d Amsterdam UMC, University of Amsterdam, Department of Nephrology, Amsterdam, the Netherlands; e Amsterdam institute for Immunology and Infectious disease, Amsterdam, the Netherlands; f Dianet Dialysis Centre, Amsterdam, the Netherlands; g Emma Children's Hospital, Amsterdam University Medical Center (Amsterdam UMC), University of Amsterdam, Department of Pediatric Nephrology, Amsterdam, the Netherlands; h Department of Pediatrics, University Hospital Antwerp, Belgium; i Department of Pediatrics, Sophia Children's Hospital, Erasmus MC, Rotterdam, the Netherlands,; j Child and Adolescent Psychiatry and Psychosocial Care, Emma Children's Hospital, Amsterdam UMC, Vrije Universiteit Amsterdam, Amsterdam, the Netherlands; k N=You Neurodevelopmental Precision Center, Amsterdam Neuroscience, Amsterdam Reproduction and Development, Amsterdam UMC, Amsterdam, the Netherlands; l Levvel, Center for Child and Adolescent Psychiatry, Amsterdam, the Netherlands; m Dept. of Neurology and Clinical Neurophysiology, University of Amsterdam, Amsterdam, UMC, the Netherlands; n Cognitive and Systems Neuroscience Group, Swameerdam Institute for Life Sciences, University of Amsterdam, Amsterdam, the Netherlands; o Amsterdam Brain and Cognition, University of Amsterdam, Amsterdam, the Netherlands

## Abstract

**Objective:**

Explore associations between quantitative markers of resting-state electroencephalography (EEG), severe chronic kidney disease (CKD), and neurocognition in children and young adults.

**Methods:**

We included 25 patients with CKD with an estimated glomerular filtration rate < 30 ml/min/1.73m^2^ aged 8–30 years, on different treatment modalities (pre-dialysis [*n* = 6], dialysis [*n* = 8], transplanted [*n* = 11]). EEG parameters of power spectrum analysis and functional connectivity were obtained using the Fourier transformation and amplitude envelope correlation (AEC) analysis, respectively. Neurocognition was measured using a comprehensive neurocognitive test battery. Mixed model analyses assessed relations between CKD parameters, EEG parameters and neurocognitive functions.

**Results:**

A longer time on dialysis in the past was significantly related to a higher delta and lower alpha power, which related to poorer Processing Speed and Working Memory. Patients on kidney replacement therapy had significantly higher delta AEC than pre-dialysis patients. A higher eGFR after transplantation was related to a higher delta AEC. Delta AEC was not related to neurocognitive performance.

**Conclusion:**

Abnormal high delta power may be indicative of clinically apparent encephalopathy in young patients with kidney failure. The role of increased functional connectivity as potential compensatory mechanism should be further explored.

**Significance:**

Minimizing dialysis-time by early transplantation may be advantageous in the prevention of encephalopathy in young CKD patients.

## Highlights

• Severe chronic kidney disease in children and young adults shows abnormal EEG slowing.
• Longer dialysis exposure is associated with EEG power changes and worse cognition.
• Kidney replacement therapy shows stronger delta connectivity, suggesting compensation.

## 1 Introduction

Severe chronic kidney disease (CKD) in children and young adults is associated with neurocognitive difficulties that can affect psychosocial development (Chen et al., 2018; Groothoff et al., 2002; Sophie Lijdsman et al., 2024; S. Lijdsman et al., 2022; Splinter et al., 2018; Tjaden et al., 2016). Symptoms reported by patients, such as ‘brain fog’, fatigue, and sleep problems, suggest altered cerebral function (Fletcher et al., 2022; Roumelioti et al., 2010; Strijkstra et al., 2003). While CKD-related changes in brain activity likely contribute to these problems (Abd El Naby et al., 2020; Bock et al., 1989; Gipson et al., 2007; Lai et al., 2016; Lizio et al., 2018; Lizio et al., 2023; Röhl et al., 2007), the extent to which CKD and its treatments affect brain activity, and how such alterations relate to neurocognition in young patients, remains unclear.

Resting-state electroencephalography (EEG) provides sensitive, non-invasive markers of brain function. Spectral power quantifies the strength of brain activity within canonical frequency bands (Niedermeyer and da Silva, 2005; Shinaberger, 2001). The few studies that have examined patients with severe CKD indicate that resting-state EEG in severe CKD is characterized by higher low-frequency (delta/theta) power (Balzar et al., 1986; Bock et al., 1989; Bourne et al., 1975; Lai et al., 2016; Röhl et al., 2007). These studies suggest that adults on dialysis may have the highest delta power, followed by transplanted patients, while pre-dialysis patients resemble healthy controls (Lai et al., 2016). Findings in the higher frequency bands are inconsistent, with studies reporting higher, lower, or unchanged power in alpha and beta bands in CKD (Balzar et al., 1986; Jonkman et al., 1992; Lai et al., 2016; Lai et al., 2018; Röhl et al., 2007; Sagalés et al., 1993; Spehr et al., 1977). Moreover, pediatric data on resting-state EEG power in CKD are lacking.

Beyond local power, cognition depends on large-scale network coordination (Bosman et al., 2014; Buzsáki and Vöröslakos, 2023; Singer, 2018; Vezoli et al., 2021; Vinck et al., 2023). EEG functional connectivity quantifies the synchrony of brain activity across regions, typically via power- or phase-based correlations within frequency bands, and complements spectral power measures (Arnulfo et al., 2015; Doron et al., 2012; Hipp et al., 2012). Despite this relevance, functional connectivity in CKD has not been systematically examined, and its relation to neurocognitive outcomes in young patients is unknown. Because frontal dysfunction is frequently implicated in CKD (Abd El Naby et al., 2020; Hamiwka et al., 2008; Kramer et al., 1996; Onder et al., 2007), connectivity between frontal and widespread regions is particularly relevant. We used eyes-open resting-state recordings to reduce alpha dominance and better index tonic wakeful brain activity (Klaver et al., 2023).

This study had two objectives: 1) to assess the impact of CKD parameters and treatment modality on brain activity patterns using power and functional connectivity analyses, and 2) to investigate associations between EEG parameters and neurocognitive impairments in children and young adults with CKD. Based on prior work (Balzar et al., 1986; Bock et al., 1989; Bourne et al., 1975; Lai et al., 2016; Röhl et al., 2007), CKD and its treatments are expected to primarily affect low-frequency brain activity (particularly in the delta band), which may in turn be associated with the neurocognitive impairments observed in CKD.

## 2 Methods

### 2.1 Participants

The study protocol was approved by the Medical Ethics Committee of the Amsterdam UMC (NL61708.018.17), and all procedures were performed according to the Declaration of Helsinki. Patients with severe CKD were recruited from the Amsterdam University Medical Center, Erasmus Medical Center (the Netherlands), and the University Hospital Antwerp (Belgium) under the supervision of their treating nephrologist. Interested patients received comprehensive information and were provided sufficient time to consider participation. Written informed consent was obtained from legal guardians for children under the age of sixteen, as well as from participants aged twelve years and older.

Typically, each session involved two main components conducted on the same day at the Amsterdam UMC. The first component was the EEG recording, which was performed by trained neuropsychologists and EEG technicians. The second component involved a 90-min neurocognitive assessment administered by a trained neuropsychologist in a designated assessment room. Furthermore, one week prior to these assessments, patients and/or their parents (for patients <18 years) completed the necessary online questionnaires through the KLIK portal (Haverman et al., 2013).

Inclusion criteria encompassed CKD stage 4–5 on conservative therapy (i.e. pre-dialysis therapy), undergoing dialysis, or having received a kidney transplant at least two years prior (to ensure stable kidney function), with ages ranging from 8 to 30 years. Exclusion criteria comprised: (1) established severe intellectual impairment or overt learning disability; (2) insufficient proficiency in the Dutch language; (3) inadequate hearing and visual acuity; (4) documented skull or brain abnormalities unrelated to CKD; or (5) co-existing diseases with primary or secondary central nervous system involvement that could interfere with the impact of CKD.

#### 2.1.1 Treatment subgroups

Three distinct treatment subgroups were identified among CKD patients: (1) a pre-dialysis group (*n* = 6) with a current estimated glomerular filtration rate (eGFR) below 30 ml/min/1.73m^2^ on conservative treatment at the time of the assessment, (2) a dialysis group (*n* = 8); and (3) a transplanted group (*n* = 11) consisting of patients with a stable, functioning kidney graft for at least two years, with an eGFR above 30 ml/min/1.73m^2^. CKD patients who had previously undergone kidney transplantation, but had an eGFR below 30 at the time of assessment were allocated to either the pre-dialysis group (n = 1) or the dialysis group (n = 1), based on their current treatment modality.

### 2.2 Measurements

#### 2.2.1 Socio-demographic and CKD parameters

We collected socio-demographic parameters including age, sex, and parental educational level using an online custom-made questionnaire. Parental educational level was categorized into three groups: (1) low education (primary education, lower vocational education, lower and middle general secondary education); (2) middle education (middle vocational education, higher secondary education, pre-university education); and (3) high education (higher vocational education, university) (Onderwijsindeling, 2016).

CKD parameters included age at diagnosis of severe CKD, current eGFR, duration of severe CKD (expressed as a % of life, calculated as [age at assessment – age at eGFR<30 ml/min/1.73m^2^] / calendar age), dialysis duration (% of life, calculated as duration of current and/or prior dialysis exposure / calendar age), and time since successful transplantation (% of life, calculated as [age at assessment – age at transplantation date & eGFR>30 ml/min/1.73m^2^] / calendar age). For patients currently on hemodialysis, we recorded EEG on a non-dialysis day. Considering significant fluctuations in eGFR before and after dialysis, the eGFR of patients undergoing dialysis was conservatively set at 10 (Janmaat et al., 2017; Okuda et al., 2019).

#### 2.2.2 EEG

##### 2.2.2.1 EEG acquisition

We obtained EEG recordings using BrainQuick SD LTM 64 recorder (Micromed, Mogliano Veneto, Italy). The international 10–20 system was employed to position the following 19 electrodes: Fp1, Fp2, F7, F3, Fz, F4, F8, T3, C3, Cz, C4, T4, T5, P3, Pz, P4, T6, O1, and O2. The ground electrode was positioned at the midline, while linked-ear electrodes served as a reference (retained for analyses; limitations of this reference schema are addressed in the Discussion). The recordings were conducted at a sampling frequency of 256 Hz. Participants were comfortably seated on a bed during the session. The EEG data were acquired for 180 s with eyes closed, followed by 180 s with eyes open. The eyes-closed segment was acquired as part of the broader resting-state EEG protocol; however, only the eyes-open segment was analyzed in the present study because it reduces eye-closure-related alpha dominance and better indexes tonic wakeful brain activity. All subsequent analyses used the eyes-open condition to reduce alpha dominance and index tonic wakefulness. During the eyes-open condition, participants were instructed to fixate on a fixation point in the room. After this, patients also performed a 20-min attention task while EEG was recorded, which is reported elsewhere (Lijdsman et al., under review).

##### 2.2.2.2 EEG pre-processing, and analyses of power and amplitude envelope correlation (AEC)

Signal preprocessing, power spectral analysis, and AEC analysis were conducted using FieldTrip (http://www.fieldtriptoolbox.org) (Oostenveld et al., 2011) implemented in MATLAB R2018b (Mathworks, 9.5.0.12298439). All subsequent analyses were performed on the EEG recordings acquired with eyes open. To eliminate power line artifacts, a digital notch filter was applied (Arnts et al., 2020). For each recording session, we separated the continuous 180-s recording into epochs of 1 s each. These epochs were visually inspected to discard ocular and muscular artifacts. No additional high-pass filter was applied to preserve low-frequency content relevant to delta; blinks and slow drifts were addressed only by visual rejection of contaminated epochs. We obtained 159 ± 10 artifact-free epochs per subject, which were used for subsequent analyses. The spectrally decomposed Fourier coefficients of each epoch (per electrode) were obtained by applying a discrete fast Fourier transform (FFT) to the segmented trials.

**Fig. 1:**
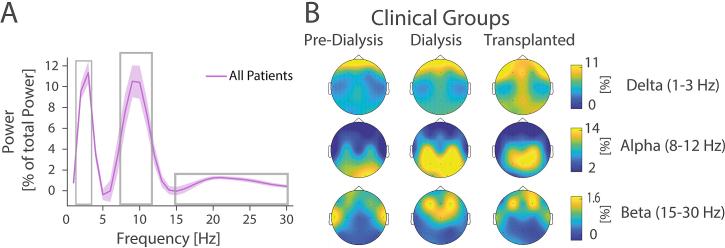
Illustration of power (% of total power) in the CKD sample. Panel A: Group-averaged power spectrum of all CKD patients, with boxes indicating the predefined frequency bands used in subsequent analyses. Panel B: Topographic maps showing the scalp distribution of power within the delta, alpha, and beta bands.

We performed spectral power analysis to quantify band-limited brain activity (i.e.*,* amplitude). Because scalp EEG spectra contain both oscillatory (periodic) peaks and an aperiodic 1/f background, we used irregular-resampling auto-spectral analysis (IRASA) to separate these components (Wen and Liu, 2016). This step was included to improve the interpretability of band-power estimates, particularly for low-frequency bands, because conventional spectral estimators quantify total power but do not distinguish oscillatory activity from broadband aperiodic background structure. In brief, IRASA estimates the aperiodic component by irregularly resampling the signal: this procedure shifts narrowband oscillatory peaks while leaving the fractal/aperiodic component largely unchanged. We first computed the original power spectrum from Hanning-tapered Fourier coefficients and then derived the oscillatory component by subtracting the IRASA-estimated aperiodic spectrum from the original spectrum. Absolute power was then normalized as relative power (percentage of total power between 1 and 30 Hz) and averaged per electrode. Based on the resulting spectrum, we focused on the three prominent frequency bands observed in the data (Fig. 1A): delta (1–3 Hz), alpha (8–12 Hz), and beta (15–30 Hz).

Topography maps were generated for each frequency band by interpolating the power (% of total power) per frequency band (per patient) across the topographical distribution of the electrodes (Fig. 1B).

We employed Amplitude Envelope Correlation (AEC) analysis to quantify functional connectivity between electrodes (Hipp et al., 2012). For each band, per-epoch band power values were extracted at each electrode and at a frontal composite seed (average of Fp1 and Fp2). We then computed the Pearson correlation (r) across epochs between each electrode's band-power series and the frontal seed's series. This approach was chosen as previous evidence showed that frontal brain activity may be particularly affected during CKD (Abd El Naby et al., 2020; Hamiwka et al., 2008; Kramer et al., 1996; Onder et al., 2007). Subject-level AEC was obtained by averaging these correlations across electrodes. AEC thus yielded a hypothesis-driven summary measure of *frontal-to-the rest of the brain* functional connectivity for each frequency band, chosen to capture global frontal coupling while reducing dimensionality in this modest sample. Higher AEC values indicate stronger functional connectivity. For subsequent analyses, we focused on delta, alpha, and beta bands.

#### 2.2.3 Neurocognitive functioning

##### 2.2.3.1 Neurocognitive test battery

We used a short form of the Wechsler (Adult) Intelligence Scale (WISC/WAIS)-III to estimate age-standardized full-scale Intelligence Quotient (eFSIQ; M = 100, SD = 15) from Block Design and Vocabulary subtests, which has demonstrated high validity and reliability in estimating IQ (Sattler, 2001). A comprehensive neurocognitive test battery was administered to assess a wide range of specific neurocognitive functions (for specifications, see (S. Lijdsman et al., 2022)). Age-standardized scaled scores were calculated for these neurocognitive tests by comparing each participant's individual score to a healthy norm group. These scaled scores were subsequently transformed into z-scores, where lower scores indicate poorer performance.

To reduce the number of neurocognitive outcome measures, we performed data reduction using principal component analyses with Varimax rotation on age-standardized z-scores derived from the neurocognitive tasks (for a detailed explanation of the procedure, see (S. Lijdsman et al., 2022)). Based on factor loadings the neurocognitive components were labeled as follows: (1) processing speed and working memory; (2) fluency; (3) verbal memory; (4) processing speed, switch, and control; (5) switching.

### 2.3 Statistical analyses

Analyses on socio-demographic and clinical characteristics were performed using SPSS 28.0 (IBM Corp., 2021). Normality and outliers (±3 interquartile ranges below/above the lower or upper quartile), were assessed for independent and dependent variables. Outliers were adjusted through a technique known as winsorizing (Field, 2013). Winsorizing was implemented by rescaling extreme values to the nearest non-outlier value. To compare treatment subgroups, Analysis of Variance (ANOVA) was used to assess differences in age, sex, parental educational level, and CKD parameters. Socio-demographic variables that exhibited a significant association with a specific outcome measure were included as covariates in the relevant analyses.

Analyses on power (% of total power) and AEC (r) for each pre-defined frequency band (i.e., delta, alpha, beta) were performed using the *HypothesisTest* package v0.10.2 implemented in Julia (version 1.5.3, (Bezanson et al., 2017)). For AEC, group differences between young CKD patients receiving kidney replacement therapy (KRT) and those not receiving KRT were assessed using Welch's *t*-test or Mann-Whitney *U* tests, depending on normality and sample size. For power, treatment subgroup spectra were inspected descriptively, while inferential analyses focused on generalized mixed models (GLMMs) relating CKD parameters to power in the predefined frequency bands. To examine the associations between CKD parameters (age at diagnosis of severe CKD, current eGFR, severe CKD duration, dialysis duration, and time since successful transplantation) and EEG measures, we used GLMMs across patients, with random effects for patients and electrodes in the power analyses and for patients only in the AEC analyses (Johnson et al., 2015; Tuerlinckx et al., 2006). Because our main hypothesis concerned CKD-related slowing of brain activity and abnormal low-frequency dynamics, delta power and delta AEC were designated a priori as primary EEG outcomes. Alpha and beta bands were additionally analyzed to provide a broader spectral characterization, but these analyses were considered exploratory secondary outcomes given the more limited a priori basis and the need to constrain the number of principal comparisons in this modest sample.

For EEG parameters showing a significant association with CKD (association with CKD-parameter or difference between treatment groups), we also examined the relationship between that EEG measure and neurocognitive components with previously observed sensitivity for CKD in young patients (specifically, eFSIQ and Processing Speed & Working Memory (S. Lijdsman et al., 2022)). We employed a GLMM to investigate this relationship, considering patients and electrodes as random effects (Johnson et al., 2015; Tuerlinckx et al., 2006). Interaction effects between CKD parameters and EEG parameters (i.e. power and AEC) were also included as predictors in the GLMM. The statistical significance was tested using a likelihood ratio test under a χ^2^ distribution.

All statistical tests were two-sided, with a significance level (alpha) set at 0.05. Given the modest sample size and the combination of predefined primary outcomes with exploratory secondary analyses, we did not apply a formal correction for multiple comparisons across the full set of statistical tests. Instead, we limited the number of comparisons by defining delta power and delta AEC as primary EEG outcomes, treating alpha and beta analyses as exploratory secondary analyses, reducing neurocognitive outcomes by principal components analysis, and restricting EEG-neurocognitive analysis to EEG parameters and neurocognitive outcomes that had shown significant CKD-related effects in prior analyses.

To facilitate the interpretation of the multimodal statistical framework, an overview of the variables included in the analyses and their role in the models is provided in Supplementary Table 1.

Finally, for simple between-group comparisons, we reported Cohen's *d* effect sizes where appropriate, and interpreted them as small (*d* < 0.5), medium (0.5 ≤ *d <* 0.8), or large (*d ≥ 0.8)* (Cohen, 1988). For the main EEG analyses, inference was based on generalized linear mixed models rather than Cohen's *d*.

## 3 Results

### 3.1 Socio-demographic and clinical characteristics

Twenty-five patients participated in the study, recruited from Amsterdam UMC (*n* = 22), Erasmus Medical Centre (*n* = 1), and University Hospital Antwerp, Belgium (n = 2). All patients completed both EEG resting-state measurements and neurocognitive assessment. The socio-demographic and clinical characteristics of the sample are shown in Table 1. Age at diagnosis was significantly higher in the dialysis group compared to the transplanted group (*p* = 0.032, *d* = 1.34). Consistent with expectations, the treatment subgroups differed in terms of eGFR, blood urea levels, and time since transplantation (see Table 1). No significant differences were observed in the comparisons of socio-demographic and other CKD parameters, and additional confounding analyses revealed no significant associations between sociodemographic variables and outcome measures.

**Table 1:** Demographic and clinical characteristics of the study population.

	CKD group	CKD treatment group			Statistics	
		Pre-dialysis	Dialysis	Transplanted	p	contrasts
n	25	6	8	11		
Age	21.1 (9.1–30.5)	16.6 (10.2–26.6)	23.0 (9.6–27.1)	18.8 (9.1–10.5)	0.560	
Male	*n* = 16 (64%)	*n* = 5 (83.3%)	*n* = 4 (50.0%)	*n* = 7 (63.6%)	0.471	
Educational level parents^a^	2.0 (1.0–3.0)	2.0 (2.0–3.0)	2.0 (1.0–3.0)	2.0 (1.0–3.0)	0.579	
Age at CKD diagnosis (years)	14.8 (0.0–24.7)	16.0 (1.9–24.7)	19.9 (9.3–24.0)	7.6 (0.0–22.8)	**0.032**	D > Tx
Primary disease (*n*)^b^						
CAKUT^1^	7	2	0	0	5	
Renovascular^2^	3	0	0	0	3	
Cortical necrosis^3^	3	0		2	1	
Acquired Glomerulopathy^4^	3	1		2	0	
Inherited nephropathy^5^	6	2		3	1	
Other & unknown cause^6^	3	1		1	1	
eGFR (ml/min/1.73 m^2^)^c^	28.0 (10.0–90.0)	22.4 (11.3–29.0)	10.0 (10.0–10.0)	51.2 (31.0–90.0)	**<0.001**	Tx > PD & D
Urea (mmol/L)^d^	14.9 (5.1–28.8)	16.2 (14.9–20.3)	21.4 (16.8–28.8)	8.2 (5.1–14.5)	**<0.001**	D > PD > Tx
Duration severe CKD (% of life)	11% (0–81%)	3% (0–81%)	11% (3–45%)	16% (4–78%)	0.632	
Ever treated by dialysis (n)^e^	15	2	8	5		
Hemodialysis	6	2	3	1		
Peritoneal dialysis	8	-	5	3		
Both	1	-	-	1		
Duration dialysis (% of life)	1% (0–49%)	0% (0–3%)	4% (1–40%)	0% (0–49%)	0.422	
Ever treated by renal transplantation (n)	14	1	2	11		
Pre-emptive	6	-	-	5		
Non-pre-emptive	8	1	2	6		
Time since renal transplantation (% of life)	6% (0–75%)	0% (0–6%)	0 (0–10%)	22%(13–75%)	**<0.001**	Tx > PD & D

Note: Values are displayed as median (range), unless otherwise indicated. Abbreviations: CKD = chronic kidney disease; GFR = glomular filtration rate.

^a^ a 1.0 = low education, 2.0=middle education, 3.0=high education.

CKD parameters were extracted from the patient’s medical file, specifications are as follows:

^b^ Primary diseases: ^1^urethral valves (*n*=7), ^2^atypical hemolytic uremic syndrome (HUS) (*n*= 1), malignant hypertension (*n*=2), ^3^due to asphyxia (*n*=1), due to septicemia (*n*=2); ^4^primary Focal Segmental Glomerulosclerosis (FSGS) (*n*=1), Anti-Neutrophilic Cytoplasmic Autoantibodies (ANCA) vasculitis (*n*=1), LE-nephritis (v= 1); ^5^NPHP1 mutation (*n*=1), Autosomal dominant polycystic kidney disease (ADPKD) (*n*=1), Alport’s syndrome (*n*=1), inherited FSGS due to INF2 mutation (*n*=2), Pax-2 mutation (*n*=1); ^6^Tubulointerstitial Nephritis (*n*=1), unknown cause (*n*=2).

^c^ creatinine levels were obtained closest to the date of study participation (range: -51 days to +4 days relative to participation date), of which the eGFR was calculated using the Schwarz formula for patients aged under 18 years (Schwartz et al., 2009), while the abbreviated Modification of Diet in Renal Disease formula was utilized for patients aged 18 and above (Levey et al., 2007). Considering significant fluctuations in eGFR before and after dialysis, the eGFR of patients undergoing was conservatively set at 10 (Janmaat et al., 2017; Okuda et al., 2019).

^d^ urea blood levels were obtained closest to the date of study participation (range: −51 days to +4 days relative to participation date).

### 3.2 EEG power spectrum analysis: Brain activity across frequency bands

Fig. 1A shows the group-averaged power spectrum of CKD patients, normalized to total power (1–30 Hz). The power spectrum exhibits three peaks, corresponding to delta, alpha, and beta frequency bands. In healthy individuals, a prominent delta peak is not typically observed (Barry et al., 2007; Niedermeyer and da Silva, 2005), suggesting that the delta peak seen in our patients reflects abnormal slowing.

To investigate the spatial distribution of power across treatment groups, we employed topographic maps for each predefined frequency band (Fig. 1B). In general, the spatial distribution was similar across treatment subgroups. Specifically, frontal electrodes showed prominent delta power, central-parieto-occipital electrodes showed prominent alpha power, and frontotemporal electrodes showed prominent beta power.

**Fig. 2:**
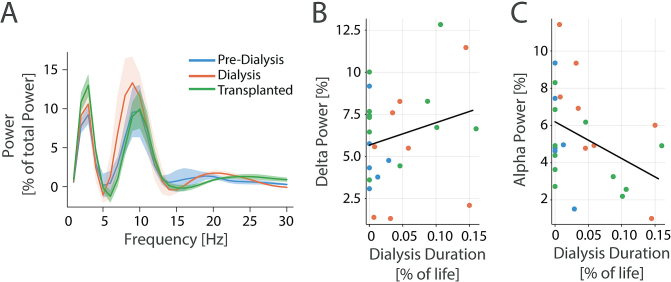
Panel A: Group-averaged power spectrum for each treatment subgroup: pre-dialysis (blue), dialysis (red), and transplanted (green). This panel provides a descriptive overview of subgroup spectra. Shaded areas indicate ± 1 standard error of the mean (SEM) around the group mean. Panels B and C show the associations between dialysis duration (% of life) and relative power (% of total power) in specific frequency bands, demonstrating that longer dialysis duration was associated with higher delta power (Panel B) and lower alpha power (Panel C). Dialysis duration reflects cumulative lifetime exposure to dialysis, including prior dialysis episodes. Therefore, two patients currently classified in the pre-dialysis group had values slightly above zero. (For interpretation of the references to colour in this figure legend, the reader is referred to the web version of this article.)

Fig. 2A shows the power spectrum across treatment subgroups. The results of the mixed model analysis indicated that dialysis duration was significantly related to power in the delta and alpha frequency bands (Table 2). Specifically, longer dialysis duration was associated with higher delta power (Fig. 2B, *p* = 0.02, χ^2^(1) = 4.8, likelihood ratio test) and lower alpha power (Fig. 2C, *p* = 0.01, χ^2^(1) = 6, likelihood ratio test). No other significant associations between CKD parameters and power were identified.

**Table 2:** Associations between CKD parameters and EEG parameters.

EEG parameters	predictors	statistics		
		*B (SE)*	*z*	*p*
Delta power [% of total power]	Age at CKD diagnosis	−0.21 (0.13)	−1.69	0.098
	eGFR	0.03 (0.02)	1.46	0.144
	Duration of severe CKD	−6.81 (3.73)	−1.82	0.068
	Dialysis duration	23.10 (10.31)	2.24	**0.025**
	Time since renal transplantation	−1.81 (3.59)	−0.50	0.615
Delta AEC [R]	Age at CKD diagnosis	0.01 (0.01)	0.74	0.458
	eGFR	0.00 (0.00)	2.17	**0.030**
	Duration of severe CKD	0.32 (0.22)	1.48	0.139
	Dialysis duration	0.97 (0.61)	1.60	0.110
	Time since renal transplantation	−0.15 (0.21)	−0.71	0.478
Alpha power [% of total power]	Age at CKD diagnosis	−0.01 (0.11)	−0.10	0.920
	eGFR	−0.02 (0.02)	−0.98	0.327
	Duration of severe CKD	2.46 (3.41)	0.72	0.471
	Dialysis duration	−24.74 (9.42)	−2.63	**0.009**
	Time since renal transplantation	−1.70 (3.28)	−0.52	0.606
Beta power [% of total power]	Age at CKD diagnosis	0.04 (0.02)	1.88	0.060
	eGFR	−0.00 (0.00)	−0.14	0.888
	Duration of severe CKD	0.24 (0.68)	0.36	0.722
	Dialysis duration	1.10 (1.87)	0.59	0.555
	Time since renal transplantation	0.68 (0.65)	1.05	0.029

Note. Abbreviations: CKD = chronic kidney disease; EEG: electroencephalogram; eGFR = estimated glomular filtration rate; AEC = amplitude envelope correlation; SE = standard error.

### 3.3 AEC analysis: Frontal functional connectivity

**Fig. 3:**
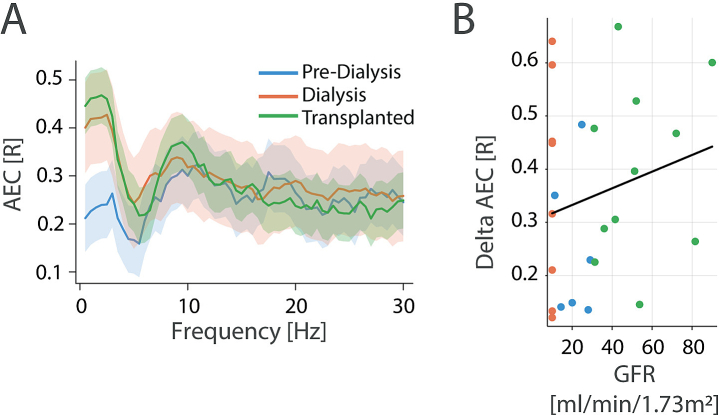
Panel A shows the AEC spectrum, expressed as correlation coefficients (r), for the three treatment subgroups: pre-dialysis (blue), dialysis (red), and transplanted (green). Panel B shows that higher eGFR is associated with higher delta AEC; each individual patient is represented by a dot, colored according to treatment subgroup. (For interpretation of the references to colour in this figure legend, the reader is referred to the web version of this article.)

**Fig. 4:**
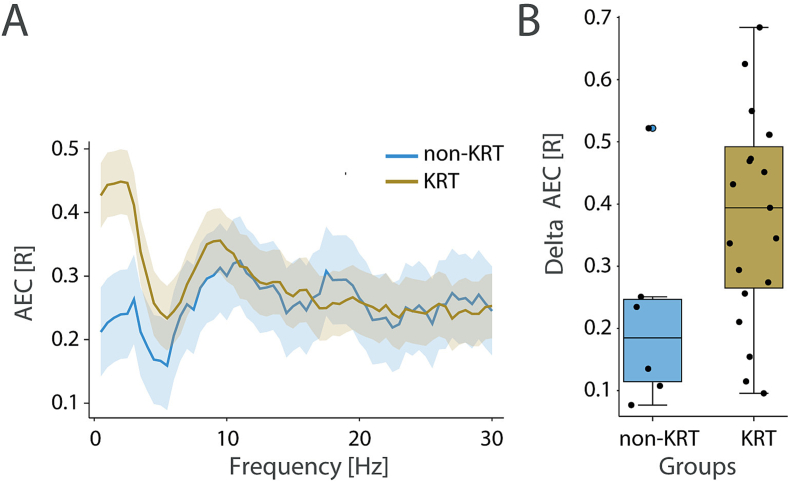
Panel A shows the AEC spectrum, expressed as correlation coefficients (r; shaded area = SD), in the non-kidney replacement therapy (non-KRT) and kidney replacement therapy (KRT) groups. Panel B shows boxplots and the statistical comparison of delta AEC between non-KRT and KRT groups.

The AEC spectrum for the three treatment subgroups is shown in Fig. 3A. Visual inspection revealed noticeable differences between subgroups only in the delta frequency band. Because the AEC spectrum displayed a similar pattern for the dialysis and transplanted groups (see Fig. 3A), we merged these groups into a kidney-replacement therapy (KRT) group. The non-kidney replacement group (non-KRT) comprised the pre-dialysis patients. Fig. 4A illustrates the AEC spectrum for the non-KRT and KRT groups, showing higher AEC values in the delta band for KRT compared to non-KRT group. The statistical comparison of the AEC in the delta frequency band showed a significant difference between groups (Mann-Whitney *U* test, U = 25, ranksum = [46,279], *p* = 0.042), with the KRT group exhibiting higher delta AEC than the non-KRT group (Fig. 4B). No significant differences were observed in the alpha or beta bands.

The mixed model analysis revealed that higher eGFR was associated with higher delta AEC (Table 2, see Fig. 3B). No significant associations between other CKD parameters and delta AEC were found (see Table 2).

In summary, our analyses suggest that KRT patients have stronger frontal functional connectivity in the delta band compared to non-KRT patients. Patients with higher kidney function (eGFR), which in our sample mainly occurred in successfully transplanted patients, also exhibited stronger frontal delta connectivity.

### 3.4 Association between CKD treatment, EEG parameters, and neurocognitive performance

Table 3 shows the significant results from the mixed model analyses examining associations between CKD parameters, EEG parameters (delta power, alpha power, delta AEC), and neurocognitive domains previously shown to be sensitive to CKD (i.e. eFSIQ and Processing Speed & Working Memory). We found no direct associations between EEG measures and neurocognitive outcomes. However, there were significant interaction effects between dialysis duration and both delta and alpha power on Processing Speed & Working Memory performance. For delta power, this interaction indicated a stronger negative association between dialysis duration and Processing Speed & Working Memory performance in patients with higher delta power. Regarding alpha power, the interaction effect indicated a stronger negative association between dialysis duration and Processing Speed & Working Memory performance in patients with lower alpha power. No other significant associations were observed.

**Table 3:** Significant mixed model analyses investigating the relation between CKD parameters, EEG parameters and neurocognitive functioning.

Neurocognitive domain	CKD and EEG parameters	statistics			Likelihood test			
		*B (SE)*	*z*	*p*	*df*	*deviance*	*χ*^*2*^	*p*
Processing Speed & Working Memory	Dialysis duration [% of life] x Delta power	−1.37	−2.19	**0.028**	6	52.2	17.2	<0.001
	Dialysis duration [% of life] x Alpha power	2.86	3.26	**0.001**	6	48.1	21.5	<0.001

*Note.* Abbreviations: CKD = chronic kidney disease; SE = standard error.

Non-significant results are not displayed, but can be made available upon request.

In summary, our analyses suggest that longer dialysis duration in CKD is related to poorer Processing Speed and Working Memory performance, particularly for patients with higher delta power and lower alpha power.

## 4 Discussion

This study investigated the impact of severe CKD and treatment modality on brain activity patterns, assessed with EEG power and functional connectivity analysis, and examined their potential associations with neurocognitive performance in children and young adults. We showed that longer dialysis duration was associated with an abnormal resting-state pattern characterized by higher delta power and lower alpha power, which in turn related to poorer processing speed and working memory. Patients receiving kidney replacement therapy (dialysis or kidney transplantation) showed stronger functional connectivity in the delta band than pre-dialysis patients. In addition, higher eGFR –observed mainly in our successfully transplanted patients— was specifically associated with stronger delta functional connectivity.

Our findings indicate that severe CKD and prior exposure to prolonged dialysis may induce a shift towards slower brain activity (i.e., a redistribution of power from the alpha to the delta band), consistent with EEG patterns previously reported in children and adults with CKD (Abd El Naby et al., 2020; Balzar et al., 1986; Florea et al., 2021; Lai et al., 2016; Lizio et al., 2023; Röhl et al., 2007). The dominance of delta power observed in our cohort likely reflects abnormal brain activity, as healthy individuals typically lack a delta peak in resting-state EEG (Barry et al., 2007; Niedermeyer and da Silva, 2005). This pattern may be relatively specific to CKD and other disorders with metabolic encephalopathy (Britton et al., 2016; Faigle et al., 2013), as prior research has shown that delta power dominance distinguish CKD patients from those with Alzheimer's disease or cerebrovascular disease (Lizio et al., 2018; Lizio et al., 2023). Interestingly, we did not find evidence of power differences between the dialysis and successfully transplanted subgroups. However, longer dialysis duration was associated with a greater shift from alpha to delta power. This suggests that prolonged time on dialysis may lead to persistent, and possibly irreversible, alterations in brain activity in young CKD patients.

Slowing of brain activity may affect neurocognitive performance, but specific data on resting-state power across specific frequency bands in young CKD patients and its association with neurocognitive functioning have been lacking. Our findings revealed an indirect association: more pronounced slowing, related to a prolonged history of dialysis therapy, was related to slower processing speed and poorer working memory. This result is consistent with studies in older CKD patients, which also report similar slowing in those with mild cognitive impairment (Lizio et al., 2018; Lizio et al., 2023). Together, these findings provide new evidence for CKD-related mechanisms that may underlie poorer neurocognitive performance in young patients with severe CKD.

To date, functional connectivity has not been systematically investigated in CKD, and future studies are needed to replicate our observations. Pending such replications, our findings suggest that patients receiving kidney replacement therapy show stronger delta-band connectivity between frontal areas and the rest of the brain, and that better kidney function (as in our successfully transplanted patients with the highest eGFR) is also associated with stronger delta connectivity. Stronger delta functional connectivity has been reported in other encephalopathies, including Alzheimer's disease and psychiatric disorders (Cattarinussi et al., 2023; Koenig et al., 2005; Schnitzler and Gross, 2005). The exact clinical implication of delta functional connectivity is not yet fully understood, but in other brain disorders, such as consciousness disorders, stronger delta connectivity has been linked with better functioning (e.g., higher level of consciousness in this group of patients) and has been proposed to reflect a compensatory mechanism (Babiloni et al., 2018; Bourdillon et al., 2020; Cattarinussi et al., 2023). We therefore speculate that the stronger delta connectivity observed in our KRT patients and in patients with better kidney functioning (higher eGFR) may reflect an adaptive response that partly offsets the neurocognitive impact of earlier dialysis-related shifts in alpha/delta power. This possibility could also explain why we did not observe direct associations between EEG parameters and neurocognitive performance. Our speculation is further supported by the finding that delta power was most prominent in frontal regions and that stronger delta connectivity was measured between frontal and most posterior brain areas.

### 4.1 Pathophysiology of EEG abnormalities in young CKD patients

We hypothesize that the high delta power observed in our patients may represent an early sign, and potential biomarker, of (uremic) encephalopathy in severe CKD. Several dialysis-related factors may contribute to power slowing and neurocognitive impairment, including acute volumetric and osmotic shifts and exposure to high levels of uremic toxins, particularly middle-molecule or protein-bound uremic toxins that accumulate and remain uncleared during dialysis, such as indoxyl sulphate, p-cresyl sulfate, homocysteine (Van Sandwijk et al., 2016; Vanholder et al., 2008). In adults, some of these uremic toxins exert direct neurotoxic effects on the brain, while others act indirectly by promoting vascular damage or chronic inflammation (Li et al., 2021; Lizio et al., 2023; Van Sandwijk et al., 2016; Vanholder et al., 2008; Yeh et al., 2016). Kidney transplantation clears these uremic toxins (Van Sandwijk et al., 2016), which may help explain the stronger delta functional connectivity observed in patients with better kidney function (mainly due to successful kidney transplantation), possibly reflecting compensatory changes in functional connectivity.

### 4.2 Strengths and limitations

We recognize several limitations of this study. First, the sample size is relatively small. Severe CKD is rare in children and young adults, which also contributed to high variability in sociodemographic and clinical characteristics. Although we observed meaningful differences, these findings require cautious interpretation and independent replications. Where possible, additional analyses were performed to account for potential confounding factors and to partially address heterogeneity. Another limitation is the broad age range of the sample, spanning from children to young adults, since developmental differences may influence resting-state EEG and particularly slow-wave activity. However, the treatment subgroups were comparable in age, and additional confounding analyses did not reveal significant associations between age and the outcome measures. Although this makes age an unlikely primary explanation for the observed effects on slowing, developmental heterogeneity remains a limitation of the present study. The absence of a demographically matched control group is a further limitation, as it restricts the extent to which the observed EEG abnormalities can be interpreted relative to typical developmental patterns. In addition, our statistical analyses were restricted to frequency bands with a clear spectral peak. As a result, we focused on the delta, alpha, and beta bands, and the potential effects in the theta or gamma range may not have been detected. Band-power estimates were derived using IRASA, which we selected to better isolate oscillatory activity from the aperiodic background component. This was important for our interpretation of EEG slowing, but alternative valid spectral approaches exist, and methodological choices may influence the precise decomposition of low-frequency activity (Donoghue et al., 2020). A further methodological consideration is the choice of EEG reference. Prior work has shown that certain reference schemes, such as mastoids or average, can artifactually inflate phase-based coherence estimates (Biggins et al., 1991; Fein et al., 1988; Nunez et al., 1997). In contrast, our analyses focused on amplitude envelope correlations, which are less sensitive to these reference-related distortions. We therefore chose linked mastoids as a conventional reference, while acknowledging that alternative reference-free approaches, such as Laplacian or an estimate of the reference at infinity, may be valuable in future studies.

Another methodological limitation is that all EEG analyses were performed at the scalp-electrode level rather than at the source level. Source reconstruction could, in principle, provide more spatially specific estimates of the cortical generators underlying the observed power and connectivity effects, and might be more sensitive to regionally specific associations with neurocognitive functioning. However, the accuracy of source-level analyses depends strongly on factors such as electrode density and forward-model precision, and with the present 19-channel montage, we considered source-level estimation insufficiently reliable for the current dataset (He et al., 2018). We therefore focused on electrode-level analyses. Future studies using higher-density EEG may help determine whether more spatially resolved source-level measures strengthen the interpretation of CKD-related network alterations.

Finally, no formal global correction for multiple comparisons was applied. Instead, we used a hypothesis-driven restriction strategy to limit the number of tests. Although this approach was considered appropriate for this modest-sized study combining predefined primary outcomes with exploratory secondary analyses, it increases the need for cautious interpretation and independent replication.

A strength of this study is that, to our knowledge, it is the first to systematically investigate the relationship between CKD, EEG power, and functional connectivity, and neurocognitive performance in young CKD patients. Another strength is the multimodal design of the study, combining resting-state EEG power, functional connectivity, clinical CKD parameters, and neurocognitive assessment within the same cohort. This allowed us to examine CKD-related brain abnormalities not only at the level of spectral slowing, but also in relation to treatment history, kidney function, and cognitive performance.

### 4.3 Conclusion, clinical relevance & future directions

This study suggests that prolonged dialysis therapy may contribute to a shift towards slower brain activity, with greater severity of this shift associated with poorer information processing and working memory. Increased frontal connectivity in patients receiving kidney replacement therapy, particularly those with better kidney function, may reflect a compensatory response to earlier dialysis-related injury. Taken together, EEG abnormalities in the delta band may serve as an early marker of uremic encephalopathy in young CKD patients. We further speculate that, in addition to vascular and inflammatory factors, metabolic mechanisms such as the accumulation of protein-bound uremic toxins not fully cleared by dialysis may contribute to abnormal brain activity in this population.

The findings highlight the potential relevance of minimizing exposure to dialysis and uremic toxins, for example, early pre-emptive transplantation or intensive nightly home hemodialysis. Future studies could also benefit from within-subject pre- and post-dialysis EEG assessments, which may help distinguish acute dialysis-related effects from more persistent CKD-associated alterations in brain activity (Florea et al., 2021). In addition, although transplantation may ultimately reduce the uremic load, the present findings raise the possibility that earlier interventions could already be relevant. Besides minimizing dialysis exposure when feasible, future work may examine whether optimizing dialysis delivery and hemodynamic stability, as well as addressing modifiable factors such as sleep, physical inactivity, and vascular risk factors, can mitigate CKD-related abnormalities in brain function before transplantation (Xie et al., 2022). Prospective longitudinal studies with demographically matched controls are needed to confirm these results and to further investigate the relationship between resting-state EEG parameters and neurocognitive outcomes in young patients with severe CKD.

## Declaration of competing interest

The authors declare that they have no known competing financial interests or personal relationships that could have appeared to influence the work reported in this paper.

## Appendix A Supplementary data

Supplementary Table 1. Overview of the statistical analyses performed in the study, including predefined primary outcomes and exploratory secondary analyses.

## References

[bb0005] Abd El Naby S.A., Bahbah W.A., Kasemy Z.A., Mahmoud A.A. (2020). Neurophysiological and Neuroradiological changes in children with chronic kidney disease. Front. Pediatr..

[bb0010] Arnts H., van Erp W.S., Boon L.I., Bosman C.A., Admiraal M.M., Schrantee A., van den Munckhof P. (2020). Awakening after a sleeping pill: restoring functional brain networks after severe brain injury. Cortex.

[bb0015] Arnulfo G., Hirvonen J., Nobili L., Palva S., Palva J.M. (2015). Phase and amplitude correlations in resting-state activity in human stereotactical EEG recordings. Neuroimage.

[bb0020] Babiloni C., Del Percio C., Lizio R., Noce G., Lopez S., Soricelli A., Bonanni L. (2018). Abnormalities of resting-state functional cortical connectivity in patients with dementia due to Alzheimer’s and Lewy body diseases: an EEG study. Neurobiol. Aging.

[bb0025] Balzar E., Saletu B., Khoss A., Wagner U. (1986). Quantitative EEG: investigation in children with end stage renal disease before and after haemodialysis. Clin. Electroencephalogr..

[bb0030] Barry R.J., Clarke A.R., Johnstone S.J., Magee C.A., Rushby J.A. (2007). EEG differences between eyes-closed and eyes-open resting conditions. Clin. Neurophysiol..

[bb0035] Bezanson J., Edelman A., Karpinski S., Shah V.B. (2017). Julia: a fresh approach to numerical computing. SIAM Rev..

[bb0040] Biggins C.A., Fein G., Raz J., Amir A. (1991). Artifactually high coherences result from using spherical spline computation of scalp current density. Electroencephalogr. Clin. Neurophysiol..

[bb0045] Bock G.H., Conners C.K., Ruley J., Samango-Sprouse C.A., Conry J.A., Weiss I., David C.T. (1989). Disturbances of brain maturation and neurodevelopment during chronic renal failure in infancy. J. Pediatr..

[bb0050] Bosman C.A., Lansink C.S., Pennartz C.M. (2014). Functions of gamma-band synchronization in cognition: from single circuits to functional diversity across cortical and subcortical systems. Eur. J. Neurosci..

[bb0055] Bourdillon P., Hermann B., Guénot M., Bastuji H., Isnard J., King J.R., Naccache L. (2020). Brain-scale cortico-cortical functional connectivity in the delta-theta band is a robust signature of conscious states: an intracranial and scalp EEG study. Sci. Rep..

[bb0060] Bourne J.R., Ward J.W., Teschan P.E., Musso M., Johnston H.B., Ginn H.E. (1975). Quantitative assessment of the electroencephalogram in renal disease. Electroencephalogr. Clin. Neurophysiol..

[bb0065] Britton J.W., Frey L.C., Hopp J.L., Korb P., Koubeissi M.Z., Lievens W.E., St Louis E. (2016).

[bb0070] Buzsáki G., Vöröslakos M. (2023). Brain rhythms have come of age. Neuron.

[bb0075] Cattarinussi G., Di Giorgio A., Moretti F., Bondi E., Sambataro F. (2023). Dynamic functional connectivity in schizophrenia and bipolar disorder: a review of the evidence and associations with psychopathological features. Prog. Neuro-Psychopharmacol. Biol. Psychiatry.

[bb0080] Chen K., Didsbury M., van Zwieten A., Howell M., Kim S., Tong A., Wong G. (2018). Neurocognitive and educational outcomes in children and adolescents with CKD: a systematic review and Meta-analysis. Clin. J. Am. Soc. Nephrol..

[bb0085] Cohen J. (1988).

[bb0090] Donoghue T., Haller M., Peterson E.J., Varma P., Sebastian P., Gao R., Voytek B. (2020). Parameterizing neural power spectra into periodic and aperiodic components. Nat. Neurosci..

[bb0095] Doron K.W., Bassett D.S., Gazzaniga M.S. (2012). Dynamic network structure of interhemispheric coordination. Proc. Natl. Acad. Sci. USA.

[bb0100] Faigle R., Sutter R., Kaplan P.W. (2013). Electroencephalography of encephalopathy in patients with endocrine and metabolic disorders. J. Clin. Neurophysiol..

[bb0105] Fein G., Raz J., Brown F.F., Merrin E.L. (1988). Common reference coherence data are confounded by power and phase effects. Electroencephalogr. Clin. Neurophysiol..

[bb0110] Field A. (2013).

[bb0115] Fletcher B.R., Damery S., Aiyegbusi O.L., Anderson N., Calvert M., Cockwell P., Kyte D. (2022). Symptom burden and health-related quality of life in chronic kidney disease: a global systematic review and meta-analysis. PLoS Med..

[bb0120] Florea B., Orasan R., Budurea C., Patiu I., Demeny H., Bondor C.I., Beniczky S. (2021). EEG spectral changes induced by hemodialysis. Clin. Neurophysiol. Pract..

[bb0125] Gipson D.S., Duquette P.J., Icard P.F., Hooper S.R. (2007). The central nervous system in childhood chronic kidney disease. Pediatr. Nephrol..

[bb0130] Groothoff J.W., Grootenhuis M., Dommerholt A., Gruppen M.P., Offringa M., Heymans H.S. (2002). Impaired cognition and schooling in adults with end stage renal disease since childhood. Arch. Dis. Child..

[bb0135] Hamiwka L.D., Midgley J.P., Hamiwka L.A. (2008). Seizures in children after kidney transplantation: has the risk changed and can we predict who is at greatest risk?. Pediatr. Transplant..

[bb0140] Haverman L., van Rossum M.A., van Veenendaal M., van den Berg J.M., Dolman K.M., Swart J., Grootenhuis M.A. (2013). Effectiveness of a web-based application to monitor health-related quality of life. Pediatrics.

[bb0145] He B., Sohrabpour A., Brown E., Liu Z. (2018). Electrophysiological source imaging: a noninvasive window to brain dynamics. Annu. Rev. Biomed. Eng..

[bb0150] Hipp J.F., Hawellek D.J., Corbetta M., Siegel M., Engel A.K. (2012). Large-scale cortical correlation structure of spontaneous oscillatory activity. Nat. Neurosci..

[bb0155] Janmaat C.J., van Diepen M., Krediet R.T., Hemmelder M.H., Dekker F.W. (2017). Effect of glomerular filtration rate at dialysis initiation on survival in patients with advanced chronic kidney disease: what is the effect of lead-time bias?. Clin. Epidemiol..

[bb0160] Johnson P.C., Barry S.J., Ferguson H.M., Müller P. (2015). Power analysis for generalized linear mixed models in ecology and evolution. Methods Ecol. Evol..

[bb0165] Jonkman J., de Weerd A.W., Poortvliet D.C., Veldhuizen R.J., van Duijn H., Rozeman C.A., Laman M. (1992). Neurometrics in cerebral ischemia and uremic encephalopathy. Brain Topogr..

[bb0170] Klaver L.M.F., Brinkhof L.P., Sikkens T., Casado-Román L., Williams A.G., van Mourik-Donga L., Bosman C.A. (2023). Spontaneous variations in arousal modulate subsequent visual processing and local field potential dynamics in the ferret during quiet wakefulness. Cereb. Cortex.

[bb0175] Koenig T., Prichep L., Dierks T., Hubl D., Wahlund L.O., John E.R., Jelic V. (2005). Decreased EEG synchronization in Alzheimer’s disease and mild cognitive impairment. Neurobiol. Aging.

[bb0180] Kramer L., Madl C., Stockenhuber F., Yeganehfar W., Eisenhuber E., Derfler K., Grimm G. (1996). Beneficial effect of renal transplantation on cognitive brain function. Kidney Int..

[bb0185] Lai S., Mecarelli O., Pulitano P., Romanello R., Davi L., Zarabla A., Lai C. (2016). Neurological, psychological, and cognitive disorders in patients with chronic kidney disease on conservative and replacement therapy. Medicine (Baltimore).

[bb0190] Lai S., Molfino A., Mecarelli O., Pulitano P., Morabito S., Pistolesi V., Lai C. (2018). Neurological and psychological changes in hemodialysis patients before and after the treatment. Ther. Apher. Dial..

[b9015] Levey A.S., Coresh J., Greene T., Marsh J., Stevens L.A., Kusek J.W., van Lente F. (2007). Expressing the Modification of Diet in Renal Disease Study equation for estimating glomerular filtration rate with standardized serum creatinine values. Clin Chem.

[bb0195] Li L.C., Chen W.Y., Chen J.B., Lee W.C., Chang C.C., Tzeng H.T., Yang J.L. (2021). The AST-120 recovers uremic toxin-induced cognitive deficit via NLRP3 Inflammasome pathway in astrocytes and microglia. Biomedicines.

[bb0200] Lijdsman S., Oostrom K.J., van Sandwijk M.S., Bouts A.H., van Hoeck K., de Jong H., Groothoff J.W. (2022). Risk factors for neurocognitive impairment and the relation with structural brain abnormality in children and young adults with severe chronic kidney disease. Pediatr. Nephrol..

[bb0205] Lijdsman S., Kerklaan J., Haverman L., van Sandwijk M.S., Bouts A.H., van Hoeck K., Groothoff J.W. (2024). Neurocognitive and adaptive functioning in young patients with severe chronic kidney disease. J. Clin. Nephrol..

[bb0210] Lizio R., Babiloni C., Del Percio C., Losurdo A., Vernò L., De Tommaso M., Gesualdo L. (2018). Different abnormalities of cortical neural synchronization mechanisms in patients with mild cognitive impairment due to Alzheimer’s and chronic kidney diseases: an EEG study. J. Alzheimer’s Dis.

[bb0215] Lizio R., Lopez S., Babiloni C., Del Percio C., Noce G., Losurdo A., Gesualdo L. (2023). Resting state EEG rhythms in different stages of chronic kidney disease with mild cognitive impairment. Neurobiol. Aging.

[bb0220] Niedermeyer E., da Silva F.L. (2005).

[bb0225] Nunez P.L., Srinivasan R., Westdorp A.F., Wijesinghe R.S., Tucker D.M., Silberstein R.B., Cadusch P.J. (1997). EEG coherency. I: statistics, reference electrode, volume conduction, Laplacians, cortical imaging, and interpretation at multiple scales. Electroencephalogr. Clin. Neurophysiol..

[bb0230] Okuda Y., Soohoo M., Tang Y., Obi Y., Laster M., Rhee C.M., Kalantar-Zadeh K. (2019). Estimated GFR at Dialysis initiation and mortality in children and adolescents. Am. J. Kidney Dis..

[bb0235] Onder A.M., Lopez R., Teomete U., Francoeur D., Bhatia R., Knowbi O., Zilleruelo G. (2007). Posterior reversible encephalopathy syndrome in the pediatric renal population. Pediatr. Nephrol..

[bb0240] Onderwijsindeling S. (2016).

[bb0245] Oostenveld R., Fries P., Maris E., Schoffelen J.M. (2011). FieldTrip: open source software for advanced analysis of MEG, EEG, and invasive electrophysiological data. Comput. Intell. Neurosci..

[bb0250] Röhl J.E., Harms L., Pommer W. (2007). Quantitative EEG findings in patients with chronic renal failure. Eur. J. Med. Res..

[bb0255] Roumelioti M.E., Wentz A., Schneider M.F., Gerson A.C., Hooper S., Benfield M., Unruh M.L. (2010). Sleep and fatigue symptoms in children and adolescents with CKD: a cross-sectional analysis from the chronic kidney disease in children (CKiD) study. Am. J. Kidney Dis..

[bb0260] Sagalés T., Gimeno V., Planella M.J., Raguer N., Bartolome J. (1993). Effects of rHuEPO on Q-EEG and event-related potentials in chronic renal failure. Kidney Int..

[bb0265] Sattler J. (2001).

[bb0270] Schnitzler A., Gross J. (2005). Normal and pathological oscillatory communication in the brain. Nat. Rev. Neurosci..

[b9000] Schwartz G.J., Munoz A., Schneider M.F., Mak R.H., Kaskel F., Warady B.A., Furth S.L. (2009). New equations to estimate GFR in children with CKD. J Am Soc Nephrol.

[bb0275] Shinaberger J.H. (2001). Quantitation of dialysis: historical perspective. Semin. Dial..

[bb0280] Singer W. (2018). Neuronal oscillations: unavoidable and useful?. Eur. J. Neurosci..

[bb0285] Spehr W., Sartorius H., Berglund K., Hjorth B., Kablitz C., Plog U., Zapf K. (1977). EEG and haemodialysis. A structural survey of EEG spectral analysis, Hjorth's EEG descriptors, blood variables and psychological data. Electroencephalogr. Clin. Neurophysiol..

[bb0290] Splinter A., Tjaden L.A., Haverman L., Adams B., Collard L., Cransberg K., Groothoff J.W. (2018). Children on dialysis as well as renal transplanted children report severely impaired health-related quality of life. Qual. Life Res..

[bb0295] Strijkstra A.M., Beersma D.G., Drayer B., Halbesma N., Daan S. (2003). Subjective sleepiness correlates negatively with global alpha (8-12 Hz) and positively with central frontal theta (4-8 Hz) frequencies in the human resting awake electroencephalogram. Neurosci. Lett..

[bb0300] Tjaden L.A., Maurice-Stam H., Grootenhuis M.A., Jager K.J., Groothoff J.W. (2016). Impact of renal replacement therapy in childhood on long-term Socioprofessional outcomes: a 30-year follow-up study. J. Pediatr..

[bb0305] Tuerlinckx F., Rijmen F., Verbeke G., De Boeck P. (2006). Statistical inference in generalized linear mixed models: a review. Br. J. Math. Stat. Psychol..

[bb0310] Van Sandwijk M.S., Ten Berge I.J., Majoie C.B., Caan M.W., De Sonneville L.M., Van Gool W.A., Bemelman F.J. (2016). Cognitive changes in chronic kidney disease and after transplantation. Transplantation.

[bb0315] Vanholder R., Van Laecke S., Glorieux G. (2008). What is new in uremic toxicity?. Pediatric Nephrology (Berlin, Germany).

[bb0320] Vezoli J., Vinck M., Bosman C.A., Bastos A.M., Lewis C.M., Kennedy H., Fries P. (2021). Brain rhythms define distinct interaction networks with differential dependence on anatomy. Neuron.

[bb0325] Vinck M., Uran C., Spyropoulos G., Onorato I., Broggini A.C., Schneider M., Canales-Johnson A. (2023). Principles of large-scale neural interactions. Neuron.

[bb0330] Wen H., Liu Z. (2016). Separating fractal and oscillatory components in the power Spectrum of neurophysiological signal. Brain Topogr..

[bb0335] Xie Z., Tong S., Chu X., Feng T., Geng M. (2022). Chronic kidney disease and cognitive impairment: the kidney-brain Axis. Kidney Dis. (Basel).

[bb0340] Yeh Y.C., Huang M.F., Liang S.S., Hwang S.J., Tsai J.C., Liu T.L., Chen C.S. (2016). Indoxyl sulfate, not p-cresyl sulfate, is associated with cognitive impairment in early-stage chronic kidney disease. Neurotoxicology.

