# Supplementary material for: Resting-state EEG reveals slowing and altered functional connectivity in children and young adults with severe chronic kidney disease
Source: Clin Neurophysiol Pract. 2026 Jun 12;11:528–37. doi: 10.1016/j.cnp.2026.06.002 (PMC13330574; doi:10.1016/j.cnp.2026.06.002)
Supplement: Supplementary file 1 — Supplementary Table 1. Overview of the statistical analyses performed in the study, including predefined primary outcomes and exploratory secondary analyses. [file mmc1.docx]

# Supplementary Table 1: Overview of variables and statistical analyses across data modalities

| **Data modality** | **Variables / parameters** | **Role in analysis** | **Status** | **Statistical approach** |
| --- | --- | --- | --- | --- |
| CKD parameters | Treatment subgroup; current eGFR; age at severe CKD diagnosis; duration of severe CKD (% of life); dialysis duration (% of life); time since successful transplantation (% of life) | Predictors | Hypothesis-driven | Generalized linear mixed models for EEG outcomes; descriptive group comparisons where relevant |
| EEG power | Relative power in delta, alpha, and beta bands | Outcomes | Delta primary; alpha/beta exploratory secondary outcomes | Descriptive subgroup visualization and generalized linear mixed models with CKD predictors |
| EEG connectivity (AEC) | Frontal-to-rest-of-the-brain AEC in delta, alpha, and beta bands | Outcomes | Delta primary; alpha/beta exploratory secondary outcomes | KRT vs non-KRT group comparisons and generalized linear mixed models with CKD predictors |
| Neurocognition | eFSIQ and PCA-derived domains (e.g., processing speed, working memory, language, memory, executive function) | Follow-up outcomes | Exploratory / conditional | Follow-up analyses restricted to EEG parameters and neurocognitive domains showing significant CKD-related effects in prior analyses (eFSIQ and the domain Processing Speed and Working Memory). |
| Sociodemographic variables | Age, sex, parental educational level | Potential covariates / confounders | Secondary | Additional confounding analyses; considered when relevant to interpretation |

**Note.** Abbreviations: AEC = amplitude envelope correlations, CKD = Chronic Kidney Disease, eGFR = estimated glomerular filtration rate, eFSIQ = estimated full-scale intelligence quotient, KRT = kidney-replacement therapy, non-KRT = non-kidney replacement therapy, PCA = principal component analyses.
